# Back disorders and lumbar load in nursing staff in geriatric care: a comparison of home-based care and nursing homes

**DOI:** 10.1186/1745-6673-4-33

**Published:** 2009-12-10

**Authors:** Kathrin Kromark, Madeleine Dulon, Barbara-Beate Beck, Albert Nienhaus

**Affiliations:** 1 Institution for Statutory Accident Insurance and Prevention in the Health and Welfare Services, Pappellallee 35/37, 22089 Hamburg, Germany; 2 Forum fBB, Moränenweg 7, 22143 Hamburg, Germany

## Abstract

**Background:**

Back pain is one of the most frequent complaints in the nursing profession. Thus, the 12-month prevalence of pain in the lumbar spine in nursing staff is as high as 76%. Only a few representative studies have assessed the prevalence rates of back pain and its risk factors among nursing staff in nursing homes in comparison to staff in home-based care facilities. The present study accordingly investigates the prevalence in the lumbar and cervical spine and determines the physical workload to lifting and caring in geriatric care.

**Methods:**

1390 health care workers in nursing homes and home care participated in this cross sectional survey. The nursing staff members were examined by occupational physicians according to the principals of the multistep diagnosis of musculoskeletal disorders. Occupational exposure to daily care activities with patient transfers was measured by a standardised questionnaire. The lumbar load was calculated with the Mainz-Dortmund dose model. Information on ergonomic conditions were recorded from the management of the nursing homes. Comparisons of all outcome variables were made between both care settings.

**Results:**

Complete documentation, including the findings from the occupational physicians and the questionnaire, was available for 41%. Staff in nursing homes had more often positive orthopaedic findings than staff in home care. At the same time the values calculated for lumbar load were found to be significant higher in staff in nursing homes than in home-based care: 45% vs. 6% were above the reference value. Nursing homes were well equipped with technical lifting aids, though their provision with assistive advices is unsatisfactory. Situation in home care seems worse, especially as the staff often has to get by without assistance.

**Conclusions:**

Future interventions should focus on counteracting work-related lumbar load among staff in nursing homes. Equipment and training in handling of assistive devices should be improved especially for staff working in home care.

## Background

Back pain is one of the most frequent complaints in the population in industrialized countries - for example the 12-month prevalence of chronic back pain in Germany in 2003 was 16% in men and 22% in women (three months and longer-lasting; almost daily). 57% of men and 66% of women reported that they had suffered from back pain (of any duration or intensity) within the previous 12 months [1]. Back pain is also a very prevalent condition among health care workers. Thus, for health care workers in different specialities, the 12-month prevalence for pain in the lumbar spine has been reported as being as high as 76% and in the cervical spine as high as 60% [2-5]. The resulting disability has enormous consequences for working life both in terms of human suffering, as well as in direct and indirect economic costs from lost working days and reduced productivity [6].

There is a growing number of older people in need of health care and services [7] and it is expected that this will lead to a considerable rise in the demand for professional nursing personnel - exceedingly in nursing homes. That demand can presumably not be met in the coming decades by training adequate numbers of new staff [8]. For this reason, it is desirable that health care workers should be professionally active for long periods of their working life. One effective way to help to achieve this goal would be to encourage preventive measures to avoid diseases related to intervertebral discs, as geriatric care is regarded as being particularly stressful for the back - especially for older health care workers [9-12]. Moreover, routine data from the Institution for Statutory Accident Insurance and Prevention in the Health and Welfare (BGW) have shown that 23.5% of the reported cases of occupational diseases of the lumbar spine related to the lifting or carrying of heavy loads were working in geriatric care, showing that this is the most affected sector within the health service [13].

In 2007, a total of 572,211 health care workers were active in geriatric care, of which 31% worked in home care facilities [14,15]. In spite of its size, the group of home care nursing staff has only been examined in a few representative studies [3,16-18]. The present study accordingly records orthopaedic findings on back disorders, intensity of back complaints, physical work load and work ability in nursing staff in nursing homes and home-based care.

## Methods

The study was advertised in specialized journals, to enrol occupational physicians in the study. The interested physicians then encouraged the facilities to take part in the study. A total of 137 geriatric care facilities throughout Germany were contacted. 63% of these facilities took part in the study (68 nursing homes and 18 home care services). A total of 3390 health care workers were contacted in the participating facilities. Documents were returned by 2164 persons (64%). Complete documentation - including the findings from the occupational physicians and a questionnaire completed by the respondents - was available for 1390 persons (41%). Participation in the study was voluntary; all subjects gave informed written consent. The study was approved by the Hamburg Medical Council.

Nursing staff was divided into three job categories: registered nurses (for general care or geriatric care) with an officially recognised diploma after a three-year training, nursing aides (including assistant nurses) with at least a one-year nursing training without examination, and nursing auxiliaries with less or no formal nursing training.

41 occupational physicians performed the orthopaedic examination. They were given training by an orthopaedic specialist in the orthopaedic examination techniques, in order to standardise data collection. Data on the intensity of the back pain in the previous four weeks and on inability to work during the preceding 12 months were recorded by the physicians. The orthopaedic examination was based on the principles of the multistep diagnosis of musculoskeletal disorders (MSD) [19]. This is a graduated examination scheme for the diagnosis of musculoskeletal disease, conceived and validated for use in occupational medical examinations [20,21]. For the present study, the original MSD examination procedure was slightly modified and consisted of a basic examination (25 items) and a complementary examination (15 items) and covered the regions of the cervical and lumbar spines. The basic examination identified abnormal features of the musculoskeletal system by inspecting the volunteers when walking or standing, by testing the mobility of the joints (actively by the patient and passively by the investigator) and by palpation. The complementary examination was performed if one of five key tests (back pain, painful inclination in the area of the lumbar spine, painful percussion in the area of the lumbar spine, the Kemp sign, or sick leave in the preceding 12 months) was documented as being positive. The complementary examination included 8 tests. In the context of the study, an orthopaedic finding was rated as abnormal if one of the criteria for the cervical or lumbar spines was documented as being positive ("yes"), or lay outside the normal range.

[See additional file 1]

The study subjects had to complete a standardised questionnaire, giving information about sociodemographic and profession-related data (training, employment status, duration of nursing experience), and the frequency of load related activities combined with patient transfers. Other components of the questionnaire were:

• Work ability (WA): WA was estimated by the subjects using the Work Ability Index (WAI). For the present study, two dimensions of the WAI were selected, dimension 2 "WA with respect to the physical and psychological demands of the work" and dimension 7 "mental resources"). The values for the WAI dimensions were calculated according to standardised coding instructions [22,23].

• Pain intensity: Information was recorded on the intensity of the pain in the regions of the cervical and lumbar spines for the 4 weeks preceding the questionnaire. Pain intensity was described according to the Graded Chronic Pain Status (GCPS) on a 10-point scale [24]. For the analysis, the scale was classified into three degrees of severity: no pain to light pain (0-3); moderate pain (4-6); intense to highly intense pain (7-10) [25].

• History of back diseases: Earlier back diseases were registered by recording the utilisation of medical services because of back pain in the preceding 12 months. This included treatment in a rehabilitation program or an individual consultation with the occupational physician.

Information on working conditions - such as the number of height adjustable patient beds, technical aids (like bath lifts and mobile lifter systems) and low tech ergonomic aids such as sliding sheets, transfer belts and anti-slip mats - were recorded from a telephone interview with the facility management.

Body mass index (BMI) was calculated on the basis of self-reported data as the ratio of body weight (kg) to the square of the height (m), adjusted by a correction factor. The values for BMI were divided into three groups, <25 (normal range), 25 to <30 (overweight), and ≥30 (obese).

The Mainz-Dortmund dose model (MDD) was used for the assessment of the lumbar load for care actvities with patient transfers [26,27]. This instrument was developed to calculate cumulative forces to the lumbar spine over the entire working life in occupational groups heavily exposed to carrying or lifting. For health care workers, 12 single activities were classified as "load related activities". Reference values of the lumbar load for these activities lie between 2.9 kN and 7.3 kN, as recommended by Theilmeier [28]. The frequencies of these load related activities were calculated semiquantitatively on the basis of the individual frequency data in the questionnaire (0-4, 5-10 or more than 10 times per shift). Specific values were assigned to each category to be used in the calculation, namely 2.5, 7.5 and 12.5, respectively. The lumbar disc compression force was calculated for health care workers in full time employment from the following formula [26]:

D_r_: Lumbar disc compression force, MDD daily dose (Newton times hours, Nh)

F_i_: Compression force on L5-S1 for single activity i (N)

t_i_: Duration of daily exposure to single activity i (h)

Each act of lifting was calculated with a loading duration of 7.5s. To assess the calculated lumbar disc compression forces, standard threshold values, namely 3.5 kNh for women and 5.5 kNh for men were used. It is assumed that the risk for back disorders is increased once these reference values are exceeded [26].

### Data analysis

To compare discrete variables - like orthopaedic findings and job related characteristics - between respondents in nursing homes and home-based care, chi-squared statistics were used, as well as analysis with the Mantel-Haenszel trend test where relevant. All continuous variables were assessed for normality using the Kolmogorov-Smirnov test. As no variable was normally distributed, the non-parametric Mann-Whitney test was used. All tests were applied two-tailed, and a significance level of 0.05 was chosen. Statistical analyses were performed using the SPSS version 14.0 for Windows.

## Results

81% of the nursing staff were working in nursing homes with exclusive or predominant inpatient care and 19% in home care services. Of the nursing homes, about 50% had more than 100 patient beds, 30% between 70 and 100 beds and 20% fewer than 70 beds. The characteristics of the nursing staff for the two care settings are described in Table 1. The staff in nursing homes were older than those in home care, had on the average worked for shorter periods in the nursing profession, had less often finished nursing training with an examination and were more frequently in full-time employment. The number of patients/residents to be cared for in each shift was higher in nursing homes than in home care, as was the proportion of patients needing intensive care, though home-based staff rarely had the possibility of requesting support from another nurse for patient transfers. Nursing staff in home care assessed their general state of health, their mental resources and their ability to handle the physical and psychological demands of the job as being better than staff in nursing homes. There was no difference between nursing staff in nursing homes and home-based care with respect to duration of sick leave due to back pain and BMI (Table 2).

**Table 1:** Characteristics of nursing staff, by care setting

	Total n = 1390 %	Nursing homes n = 1126 %	Home care n = 264 %	**p-value**^**1**^
Female gender	88	87	90	>0.05
				
Age (years)				
- under 30	18	18	15	
- 30 to 49	60	59	68	
- over 50	22	23	17	<0.05
				
School educational level (years)				
- Low (≤9)	37	40	26	
- Medium (10-11)	45	43	52	
- High (≥12)	18	17	22	<0.001
				
Nursing degree				
- Registered nurse	26	20	50	
- Registered geriatric nurse	34	37	20	
- Nursing aides', assistant nurses	21	24	10	
- Nursing auxiliaries	19	19	20	<0.001
				
Full-time employment (≥ 35 h/week)	49	53	31	<0.001
				
Number of residents per shift (including those requiring intensive care in %)				
- ≤10 (≤50%)	30	26	52	
- ≤10 (>50%)	12	13	4	
- >10 (≤50%)	39	38	42	
- >10 (>50%)	19	23	2	<0.001
				
Support possible from another nurse	86	95	50	<0.001
				
Participation during the preceding 12 months in measures to prevent back disorders				
- No measure	62	63	57	
- Primary preventive measure^2^	29	27	36	
- Secondary preventive measure^3^	9	10	7	<0.05
	Median (Quartile)	Median (Quartile)	Median (Quartile)	
Years worked in the nursing profession	11 (6-20)	11 (6-19)	16 (8-24)	<0.001

^1^Chi square test for differences between nursing staff in nursing homes and home care.

^2^Participation in workplace prevention program (use of low tech ergonomic aids, back school).

^3^Treatment in a rehabilitation program for back disorders, consultation with the facility physician.

**Table 2:** Work ability (WA) and general state of health of nursing staff, by care setting

	Nursing homes n = 1126 %	Home care n = 264 %	p-value
WA in relation to the physical and psychological demands of the job			
- rather good to very good	43	57	
- intermediate to very poor	57	43	<0.01
			
Mental resources^1^			
- rather high	78	90	
- rather slight	22	10	<0.001
			
General state of health			
- good to excellent	82	91	
- less good to poor	18	9	<0.01
			
Duration of sick leave due to back pain			
- none sick leave	83	87	ns
- sick leave up to 4 weeks	13	10	
- sick leave more than 4 weeks	4	3	
			
BMI			
- <25	40	45	ns
- 25-<30	36	34	
- ≥30	24	21	

^1 ^Composed of the items "pleasure in daily work", "active and alert" and "confident for the future; related to the last 3 months.

On the day of the examination, 18% of the staff in nursing homes complained of symptoms in the lumbar spine and 11% in the cervical spine (Table 3). There was no difference between nursing staff in nursing homes and home-based care with respect to pain intensity in the lumbar spine. In the cervical spine, pain intensity was lower for staff in nursing homes. In both groups, about one fifth of all staff suffered intense to highly intense pain in the lumbar and cervical spines.

**Table 3:** Back disorders of nursing staff, by care setting

	Nursing homes n = 1126 %	Home care n = 264 %	**p-value**^**1**^
Back pain on the examination day^2^			
- in the lumbar spine	18	16	n.s.
- in the cervical spine	11	4	<0.001
			
Pain intensity in the lumbar spine in the last 4 weeks^3^			
- none to mild	55	56	
- moderate	24	24	
- intense to very intense	21	20	ns
			
Pain intensity in the cervical spine in the last 4 weeks^3^			
- none to mild	57	63	
- moderate	25	23	
- intense to very intense	18	14	<0.05
			
*Disorders in the lumbar spine*			
Schober reclination (≥ 10 cm)	10	3	<0.01
Schober inclination (< 13 cm)	31	22	<0.01
Inclination painful	11	5	<0.01
Reclination painful	11	8	ns
Kemp sign positive	17	9	<0.001
Painful arc	6	5	ns
Climbing up the legs	5	2	<0.05
Percussion painful	11	7	<0.01
Pain in heel-fall test	5	5	ns
Lateral inclination painful^4^	18	11	<0.01
Pain during the straight leg raise test^4^	32	13	<0.001
Lasèque sign^4^	2	1	ns
Heel to buttocks distance (> 29 cm)^4^	4	5	ns
Pain during the heel to buttocks distance^4^	26	13	<0.001
Pain on stretching the femur^4^	7	2	<0.01
			
*Disorders in the cervical spine*			
Lateral inclination/flexion or extension painful	16	11	<0.01
Ott index (≤31 cm)	27	24	ns
Percussion painful	6	3	<0.05
Pain in heel-fall test	<1	1	ns

^1 ^ns = non-significant.

^2 ^Taken from the medical history.

^3 ^Taken from the questionnaire (self-reported data).

^4 ^Findings on the right and/or left leg.

About 60% of nursing staff in both groups were given the more extensive tests in the complementary examination. Of the 13 orthopaedic findings in the lumbar spine, 10 were significantly more often abnormal in staff active in nursing homes than in home care (Table 3). The findings were mostly functional restrictions (including the Schober Index, the Kemp sign, and inclination and lateral inclination in the area of the lumbar spine), as well as abnormalities in the SLR test and in the heel to buttocks distance. However, these are non-pathological findings, such as muscular pain and an intense feeling of stretching. Nerve pain in the SLR test or in the heel to buttocks distance is an expression of irritation in the nerve root. This occurred in less than 1% of the nursing staff in both groups. Examination of the cervical spine showed that the mobility in the neck and shoulder region was also more often restricted in staff in nursing homes. These findings predominantly indicate functional restriction, but may nevertheless be linked to considerable impairments in quality of life and performance.

Measures for primary prevention like training in lifting techniques and use of assistive devices were more often used by home care staff. Staff in nursing homes had more often participated in rehabilitation programs (Table 1).

In 76% of the nursing homes, the ergonomic conditions of the workplace were comparatively good. These facilities were exclusively equipped with height adjustable patients beds, of which 82% were electric beds (data not shown). About 90% of the nursing staff reported that they either occasionally or almost always adjusted the height of the bed to their working procedures (Table 4). Home-based staff more rarely carried out nursing functions at the patient bed with back-protecting work practices than did their colleagues in nursing homes (Table 4). Technical lifting aids were in almost all nursing homes (more than 90%), where they were regularly used (no table). Patient lifting poles were available in about half of all nursing homes (approx. 40%). Low tech ergonomic aids were available in about a third of nursing homes. However, they were only actually used in about two thirds of the facilities in which they were present (data not shown).

**Table 4:** Back-protecting work practices at the patient bed performed by nursing staff, separated by care setting

	Nursing homes n = 1126 %	Home care n = 264 %	**p-value**^**1**^
Adjusting height of bed is occasionally/almost always performed:			
- Changing the patient's position	98	92	<0.001
- Mobilisation	93	94	ns
- Basic nursing	96	98	ns
- Technical nursing	93	94	ns
			
Push bed away from wall			
- Occasionally/almost always	91	78	<0.001
- Rarely/never	9	22	
			
Bed rail let down			
- Occasionally/almost always	98	95	<0.05
- Rarely/never	2	5	

^1 ^ns = non-significant.

**Figure 1:**
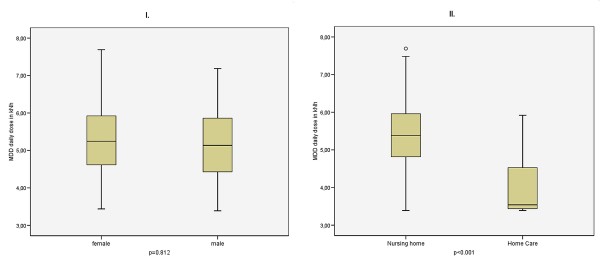
**Lumbar disc compression force of full-time nurses by gender (I) and care setting (II)**.

Ten of the twelve load related activities occurred significantly more often for staff in nursing homes, including activities such as changing the patient's position in the bed, shifting the patient up to the bed, and raising the patient from the lying position to sitting (data not shown). Calculation of the lumbar load showed that 90% of the female and 38% of the male staff were above the gender-specific reference values for the daily dose (no table). For 45% of the staff in nursing homes, the resulting lumbar load was found to be above the reference value of 5.5 kNh for men, in comparison to the figure of only 6.4% for staff in home care. The median lumbar load for staff in nursing homes was 5.4 kNh, in comparison to 3.5 kNh for home care staff (Figure 1). There was no difference between male and female staff with respect to the median lumbar load.

## Discussion

Many studies on the situation in long-term health care have concentrated on geriatric care. In the present study, we have examined the prevalence of orthopaedic findings in the back and of the lumbar load for care activities in a group of 1390 health care workers in 86 nursing homes and home care facilities. Our results show that staff in nursing homes more often gave positive orthopaedic findings and more often complained of symptoms in the cervical spine. At the same time, staff in home-based care had a more favourable impression of the condition of their general health, their work ability in relation to the demands of the work and their mental resources than did their colleagues in nursing homes. This agrees with the study by Hasson and Arnetz, which found that home care staff experienced significantly less physical and emotional strain compared with staff in nursing homes [18]. There have been few studies dealing with geriatric nursing staff as a separate group of health care workers; one of these is the NEXT study. Our findings agree with those of the NEXT study, in that staff in nursing homes more often report complaints from disorders of the cervical or lumbar spine than do staff in home care [16]. The NEXT study also reported a trend towards less favourable values for the work ability of staff in nursing homes [16,29]. When comparing the prevalence data, it should be remembered that studies on the prevalence of occupational back diseases frequently have different objectives and target variables, so that direct comparison is difficult [30]. The present study contains a systematic collection of orthopaedic findings, which is recommended in occupational medicine for the timely recognition of suspicious findings for avoiding excessive or inappropriate lumbar load [19]. In our study, nearly every second staff in elderly care had positive orthopaedic findings. However, these were not manifest orthopaedic diseases, but functional restrictions to the locomotor system. In the present study, serious findings indicating disorders related to the intervertebral bodies (nerve pain in the SLR test and pain on stretching the femur) were only made in 2% of the study group - a somewhat lower proportion than in another study with active community nurses [3]. The idea that most cases in our study were not suffering from serious diseases is supported by the fact that only every sixth staff member was on sick leave due to back problems in the year preceding the survey. Other studies investigating staff in nursing homes and general hospitals have reported comparable figures for inability to work because of back pain [31].

Height adjustable patient beds and assistive devices can both be of great help in reducing lumbar load for care activities with patient transfer in geriatric care [32-35]. Nursing homes are very well equipped with height adjustable patient beds and with technical lifting aids, though their equipment with low tech ergonomic aids must be regarded as unsatisfactory. This is in line with the NEXT study, according to which technical lifting aids were available in 78% of nursing homes in Germany and more than 90% of the staff used the available lifters [16]. Another study found somewhat lower figures of around 80% for use of technical lifters by geriatric nurses [36]. Data for the equipment with assistive devises of home-based staff were not assessed in our study; as no data have been published yet by other authors, further research is needed in this care setting. Homes-based staff is apparently more often forced to carry out patient transfers alone than happens in nursing homes. This agrees with the study by Owen and Staehler, which found that in home care a second assistant was only permitted by the agency because of the patient's weight and unwillingness to cooperate [37].

Altering the height of patient bed to ergonomic lift level is supposed to be a technique to reduce the frequency and extent of marked inclination of the trunk [38] and was adopted by staff in both care settings. Additional back-protecting work practices at the patient's bed - such as lowering bed rails before nursing actions - seem to be less well established in home-based staff. Because of lack of data about the equipment, we can only speculate about the reasons for the inadequate implementation. It is expected that space for free movement around the patient's bed is restricted under real conditions in private homes [28].

In our study, staff in nursing homes was exposed to a much higher lumbar load than staff in home care. Except for the study by Theilmeier et al. [28], which used the same exposure assessment, we found no other published study of geriatric nurses that had calculated lumbar load for the sum of daily patient transfer activities. We found higher figures of lumbar load in nursing home staff and at the same time back disorders were significantly more often found in nursing home staff than in home-based staff. Our results are consistent with previous reports indicating a dose-relation between lifting of weights or working postures with extreme forward bending and the development of lumbar disorders [39-41].

The validity of the reference values in the MDD procedure has currently been discussed in the field of occupational medicine [42] and modification has been proposed [39]. The original model is nevertheless useful to assess the lumbar load in nursing staff working in different care settings, as shown in the present study.

Calculation of the lumbar load was based on self-reported data. Misclassification is therefore possible. Nevertheless, any masking of effects in the comparison between staff in nursing homes and home care should be negligible. Data on the implementation of back-protecting work practices are also self-reported data by the respondents. For this reason, bias from subjective estimation cannot be excluded.

## Conclusions

Staff in nursing homes reports more occupational exposure on the lumbar spine than home-based staff. Furthermore, staff in nursing homes had more abnormal orthopaedic findings, a higher lumbar load and reduced values for work ability. The present data therefore support the demand for consistent and vigorous implementation of preventive measures for staff in home-based care and - to a much greater extent - in nursing homes. It appears necessary to provide advanced training to support working procedures conducive to back health which should be specifically adapted to the differences between both care settings. Additional studies are needed for more verified information about the working conditions in home care setting.

## Competing interests

The authors declare that they have no competing interests.

## Authors' contributions

KK performed the statistical analyses, interpreted the data and drafted the manuscript. MD participated in the design of the study, interpreted the data and drafted the manuscript. AN participated in the design of the study and interpreted the data. BBB participated in the coordination of the study and helped to draft the manuscript. All authors read and approved the manuscript.

## Supplementary Material

Additional file 1 **Diagnostic procedure of orthopaedic examination**. Scheme for the orthopaedic examination performed in this study according to Grifka [19]. Click here for file
