# Supplementary figures and images for: Back disorders and lumbar load in nursing staff in geriatric care: a comparison of home-based care and nursing homes
Source: J Occup Med Toxicol. 2009 Dec 10;4:33. doi: 10.1186/1745-6673-4-33 (PMC2801493; doi:10.1186/1745-6673-4-33)

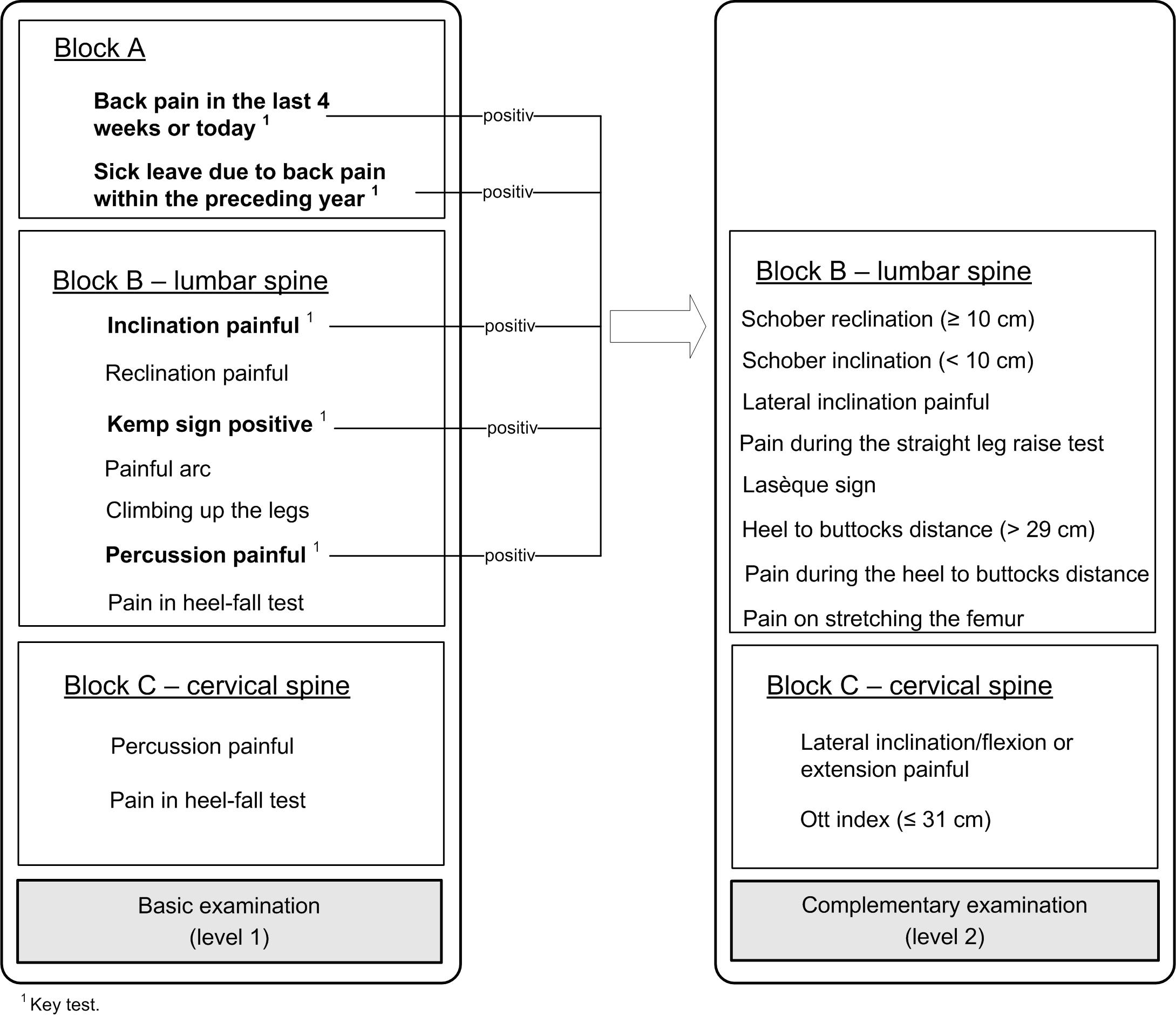

Supplement: Additional file 1 — Diagnostic procedure of orthopaedic examination. Scheme for the orthopaedic examination performed in this study according to Grifka [19]. [file 1745-6673-4-33-S1.JPEG]
